# Systematic description of an interdisciplinary multimodal pain treatment programme for patients with chronic musculoskeletal pain, using the TIDieR checklist

**DOI:** 10.1186/s13104-022-06211-z

**Published:** 2022-10-11

**Authors:** L. Breugelmans, E. Scheffer, L. W. M. E. Beckers, R. F. A. Oosterwijk, G. Nijland, R. J. E. M. Smeets

**Affiliations:** 1 Department of Rehabilitation Medicine, Care and Public Health Research Institute (CAPHRI), Faculty of Health, Medicine and Life Sciences, Maastricht University, Maastricht, Netherlands; 2 Lifestyle and Health Research Group, Healthy and Sustainable Living Research Centre, University of Applied Sciences Utrecht, Utrecht, Netherlands; 3 CIR Rehabilitation, Zwolle, Netherlands; 4 CIR Rehabilitation, Eindhoven, Netherlands; 5 Pain in Motion International Research Group (PiM), Antwerpen, Netherlands

**Keywords:** Chronic pain, Interdisciplinary care, Cognitive behavioural therapy, TIDieR, Acceptance and commitment therapy, Biopsychosocial model, Emotional awareness and expression therapy, Graded activity, Exposure in vivo, Rehabilitation

## Abstract

**Objective:**

To provide a thorough and systematic description of an interdisciplinary multimodal pain treatment programme (IMPT) for patients with chronic musculoskeletal pain (CMP), using the TIDieR checklist as a guide.

**Results:**

The main goal of the *‘Centre for Integral Rehabilitation (CIR) Excellent’* IMPT is to improve daily functioning, participation and quality of life of patients with CMP by helping them to adapt their behaviour so as to better manage their symptoms. A combination of physical and psychosocial treatment methods is employed, including Emotional Awareness and Expression Therapy (EAET), Pain Neuroscience Education (PNE), Acceptance and Commitment Therapy (ACT), graded activity, exposure in vivo, and experiential learning through physical training. The interdisciplinary treatment team comprises physiotherapists, psychologists and a physiatrist. The programme lasts 10 weeks (61 h in total) and consists of three phases: a start (Week 1), education (Weeks 2–3), and skills learning phase (Weeks 4–10). Patients come in twice a week and participate in 2–4 sessions (3–4 h) per treatment day. The programme consists of both individual (physical and mental coaching) and group sessions (education, movement and behaviour outdoors/indoors). Individualisation through personal goal-setting is an important characteristic of the treatment, as well as frequent interdisciplinary consultation between care providers.

## Introduction

The biopsychosocial model of pain lays the foundation for the widely accepted view that chronic musculoskeletal pain (CMP) is a multidimensional problem that requires interdisciplinary multimodal pain treatment (IMPT) [1–3]. Promising clinical results support the use of IMPT for patients with CMP. However, additional scientific evidence is warranted, specifically for long-term effectiveness [4]. The Centre for Integral Rehabilitation (CIR) in the Netherlands delivers a 10-week IMPT to patients with CMP. A forthcoming publication will report on the short- and long-term effects of this treatment, using a 3-year longitudinal dataset (2019–2021). Inadequate descriptions of interventions in clinical studies often impede the drawing of unambiguous conclusions about their effectiveness, comparison of study results, and reliable implementation of interventions in clinical practice [5, 6]. The TIDieR (Template for Intervention Description and Replication) checklist is a tool that addresses these shortcomings and guides the systematic description of interventions [7]. The aim of this paper is to provide a thorough and systematic description of the *CIR Excellent* treatment, using the TIDieR checklist as a guide.

## Main text

### Rationale and goals of intervention

The aim of the *CIR Excellent* IMPT is to support patients in adapting their behaviour in such a way that they can better manage their symptoms, resulting in improved functioning in personally relevant daily activities, participation and quality of life. Reduction of pain may occur as a consequence of directing attention towards actionable goals, instead of fighting pain. The patient is deliberately addressed as ‘client’ and is actively encouraged to take back control and pursue actions in line with his/her values, despite the pain. The treatment has a strong experiential component and integrates elements of a number of therapies proven to be effective for patients with CMP. Included are several elements of the recently developed Emotional Awareness and Expression Therapy (EAET), which focuses on helping patients to recognise, disclose and process intrapersonal psychological conflicts by increasing emotional awareness, improving interpersonal communication, and encouraging new, adaptive emotional reactions [8]. Another element is pain neuroscience education (PNE), which helps the patient to understand that CMP can be attributed to the brain instead of tissue damage. As a consequence, fear of perceived danger is reduced and the patient’s self-efficacy increases [9]. A third important method is Acceptance and Commitment Therapy (ACT), which is used to reduce the impact of the pain by increasing the patient’s psychological flexibility, ultimately teaching the patient to act in line with his/her personal values and goals, despite the presence of pain [10, 11]. In addition, cognitive behavioural therapy (eg. graded activity) is employed to moderate maladaptive coping behaviours. Specific attention is paid to the presence of irrational pain related fear in light of the fear-avoidance model [12]. If present, the Photograph Series of Daily Activities (PHODA) checklist [13] is used to further assess the patient’s cognitions regarding potential harmful movement and activities and the results are used to implement elements based on exposure in vivo therapy [14, 15] into the treatment programme.To facilitate behavioural change, physical training sessions based on graded activity and experiential learning are provided. Only in case of physical deconditioning or limitations in strength or coordination, or to support the patient’s individual goals, is specific training based on physiological principles used.

### Location details

CIR has six treatment centres across the Netherlands where, since 1988, secondary outpatient care for patients with CMP has been provided. All treatment centres are equipped with consultation rooms and exercise facilities. Where appropriate, sessions are performed outside.

### Eligibility criteria

**Fig. 1:**
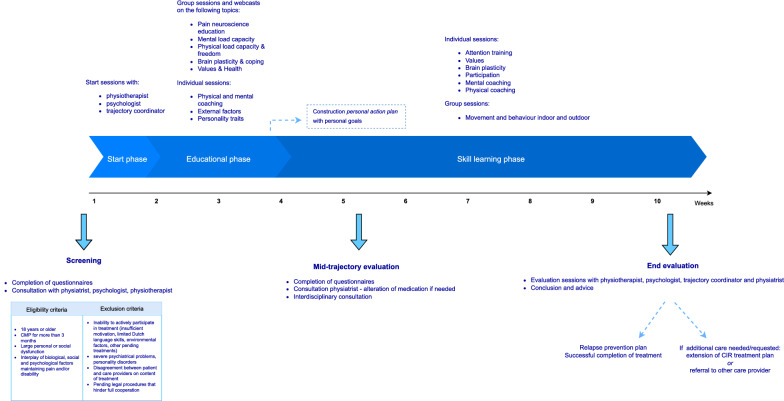
Overview of phases in the *CIR Excellent* treatment

After referral to CIR by either a general practitioner, occupational physician or other medical specialist, the patient is seen by a physiatrist, physiotherapist and psychologist. As part of this screening process, the patient has to complete a set of questionnaires and two physical performance tasks (Table 1), which are used to aid diagnostics as well as to monitor treatment progress. Based on the screening results, it is decided whether the patient qualifies for this treatment. Patients with CMP lasting longer than three months are eligible for treatment and no discrimination is made between the nature of the CMP or the affected body region(s). Additionally, there needs to be evidence of a complex interplay of biopsychosocial factors that maintains pain and limitations with regard to functioning and participation. Detailed eligibility and exclusion criteria are presented in Fig. 1.

**Table 1:** Questionnaires used for screening and evaluation purposes at intake, Week 5 (mid-trajectory evaluation), and Week 10 (end evaluation)

Name questionnaire	Abbr	Description
General CIR questionnaire	Not applicable	Records general socio-demographic characteristics, as well as symptom-related information, co-morbidity and medication levels The *follow-up questionnaire* in Weeks 5 and 10 again records the presence and strength of the symptoms, as well as the perceived effect of the treatment on pain, coping and limitations in daily functioning. In Week 10, work ability, satisfaction with the treatment and potential side effects are additionally recorded
Symptom Checklist 90^a^	SCL-90	Measures to what degree the patient suffers from 90 different physical and psychological symptoms. [16]
Pain Self-Efficacy Questionnaire	PSEQ	Measures how confident the patient is that he/she will be able to perform daily tasks despite being in pain. [17]
Psychological Inflexibility in Pain Scale	PIPS	Measures psychological inflexibility (avoidance of pain and cognitive fusion with pain). [18]
Hospital Anxiety and Depression Scale	HADS	Measures feelings of fear and depression, without looking at physical complaints. [19]
Brief Illness Perception Questionnaire	BIPQ (Dutch: IPQ-K)	Measures the cognitive and emotional representation of illness in a patient. [20]
Pain Catastrophizing Scale	PCS	Measures degree of catastrophising. [21]
Checklist Individual Strength	CIS	Measures subjective tiredness. [22]
Pain Disability Index	PDI	Measures to what degree pain prevents the patient from participating in daily activities. [23]
Twelve-Item Short Form Health Survey	SF-12	Measures physical and mental functioning. [24]
Patient-Specific Complaint	PSC (Dutch: PSK)	Measures a patient’s functional status by asking, for three examples of self-selected daily activities, to what degree the patient is limited in these activities by his/her pain complaints. [25]
6 min walk test^a^	Not applicable	Measures physical performance by recording the distance in metres the patient can walk in 6 minutes, resting and maximal heart rate, and the level of exertion at the end of the test
5 times sit to stand test^a^	Not applicable	Measures physical performance by recording the time in seconds needed for a patient to stand up five times from a chair without arms as fast as possible (twice)

All questionnaires are provided in Dutch, either as the original or a translation

^a^Only recorded at intake and Week 10

### Background and training of care providers

An interdisciplinary team consisting of a physiatrist, physiotherapists and psychologists is responsible for implementing the treatment. In addition, each patient is allocated a trajectory coordinator (a physiotherapist or psychologist) who closely monitors the patient’s complete treatment trajectory. All team members are knowledgeable about IMPT for patients with CMP, either through previous work experience or through in-house training, and have received additional (external) training on ACT. Furthermore, physiotherapists have completed training in graded activity.

### Description of procedures and activities

The duration of the treatment is ten weeks, consisting of start, education, and skills learning phases (Fig. 1).

#### Start phase (Week 1/4 h)

In the introductory group session, patients are informed about organisational aspects of the treatment, and the biopsychosocial model of chronic pain is introduced. Every patient has an individual start session with a physiotherapist, psychologist and trajectory coordinator to determine points of engagement and personal goals the patient wants to achieve.

#### Education phase (Weeks 2–3/12 h).

**Fig. 2:**
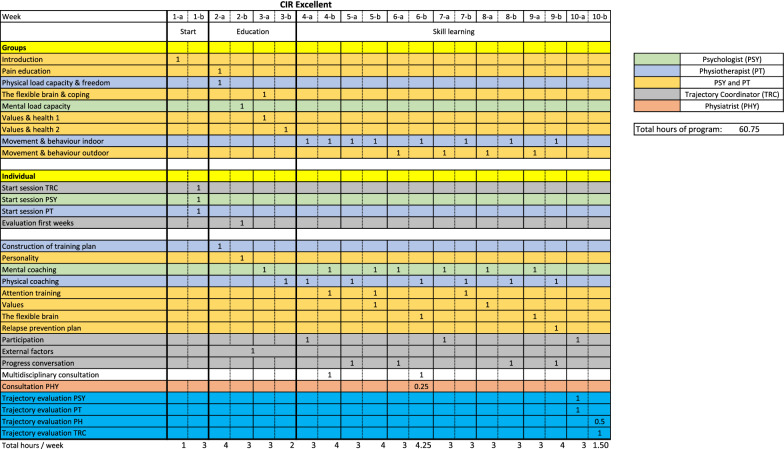
Overview of the *CIR Excellent* treatment, including timing and duration of sessions. © CIR Netherlands

Patients participate in group sessions focusing on PNE, mental and physical load capacity, brain plasticity, coping, and the importance of personal values. These sessions are accompanied by webcasts which patients are asked to watch prior to the session. In addition, patients have individual sessions of mental and physical coaching with a psychologist and physiotherapist, respectively, as well as two sessions on the influence of external factors and personality traits. A detailed schedule of all sessions, including in which phase of the programme they are provided and by which care provider, is shown in Figure 2*.* At the end of this phase, a personal action plan is drawn up in consultation with the patient. In this plan, personal patient goals are formulated in terms of *restored* or *increased participation* in daily activities, social contact, and employment.

#### Skills learning phase (Weeks 4-10/45 hours)

Consisting of (see Figure 2 for details):

- Individual sessions on topics like attention training, personal values, brain plasticity, participation, and mental coaching.
- Individual physical coaching using body-conscious and experiential movement therapy to make patients familiar with performing physical activities despite experiencing pain. Patients follow a personal training plan constructed in Week 3 and which spans the entire duration of the treatment (a time- instead of pain-contingent approach). The training plan includes functional movements that relate to (desired) daily activities using the graded activity approach.
- Group sessions on movement and behaviour indoors and outdoors: learning to use the body as an indicator of personal preferences and needs in order to promote value-based actions. Sessions comprise challenges or games in different environments with an emphasis on collaboration.

An important feature of the treatment is the interdisciplinary consultation between care providers at every stage of the process. After every session, the care provider reports in the electronic patient file (Asterisque®) on topics discussed, the patient’s progress, and any other relevant events during the session or aspects that deserve attention. Prior to the next session, this report is reviewed by the next care provider.

Every week, two interdisciplinary consultations take place where the progress of patients is discussed with the entire treatment team. Patients in Week 5 of the treatment are always scheduled to be discussed in these consultations, as well as any other patients for which the need arises. In addition, care providers discuss the progress of a patient with each other whenever they think necessary.

### Intervention frequency and timing

From Week 2 onwards, patients visit the treatment centre twice a week, participating in 2–4 sessions (3–4 h) per treatment day (see Fig. 2). During the education phase, the patient participates in 6 h of group sessions and 6 h of individual sessions. In the skills learning phase, group sessions are limited to ‘movement and behaviour’ (8 h indoors and 4 h outdoors). In addition, they have 3–4 h of individual sessions per week (27 h in total). Week 10 consists of evaluation sessions (3.5 h).

### Modes of delivery

Most sessions are provided face-to-face, both in small groups (2–5) and individually, depending on the treatment phase and session (see Table 1). Exceptions are the online webcasts in the education phase, but these are always followed by a face-to-face session on the same topic. Since the onset of the COVID-19 pandemic, sessions may be organised exceptionally via video call in specific circumstances (e.g., (potential) COVID-19 infection).

### (Informational) material used in the intervention

Care providers have access to documents that outline the topics and structure of the educational sessions and to fixed-format PowerPoint presentations. Patients receive a workbook and personal login for the online patient-portal (Asterisque®) through which they can, among other things, review background material related to the topics of the sessions, fill out the questionnaires, and view the webcasts. Patients are requested to watch the webcast prior to educational sessions and to answer the questions on the relevant topic in their workbooks, making the topic more applicable to the patient’s specific context. Furthermore, the workbook is used throughout the treatment to note goals, complete exercises, log insights gained during the sessions and to keep the patient on track.

### Evaluation of treatment progress and results

In Week 5, after the patient has completed for the second time most of the same questionnaires as at the start of the programme (see description in Table 1*)*, a mid-trajectory evaluation takes place in which the trajectory coordinator and physiatrist each discuss progress with the patient. Based on this evaluation, the physiatrist, together with the patient, can decide to alter (usually reduce) pain or other medication, or provide additional medical education. In Week 10, the final evaluation takes place with the entire treatment team participating. Prior to this, the patient has again completed a set of questionnaires. Progress is discussed with the patient and a relapse prevention plan constructed. The intention is always that the treatment be completed in 10 weeks. Where the patient goals have not been reached, but there has been considerable progress towards these goals, an extension of the treatment can be considered. The treatment team and patient discuss the feasibility of achieving the goals within the duration of the extension. If so, the length (5–20 h) and scope of the extension are precisely tailored to the patient’s needs.

### Tailoring or individualisation

All trajectories follow the same schedule and feature the same sessions in equal quantity and order. Individualisation, however, takes place on a per-session basis, so that the care provider can decide to pay more attention to certain topics if screening or evaluation identify specific needs or leverage points. Attention to individual psychological conflicts is given during, among others, the physical and mental coaching sessions, to ensure that these factors do not impede the effect of the treatment. If the patient has suffered a trauma that reinforces his/her limiting behaviours and counteracts the efficacy of the treatment, four regular sessions can be replaced by Eye Movement Desensitization and Reprocessing (EMDR) sessions, preferably early in the skills learning phase. In case of an adverse event, the therapist involved will report this to the physiatrist and medical or psychological care will be provided or sought for. A specific protocol is available in case of potential risk for suicide. Furthermore, in the follow up questionnaires, the patient is requested to report any side effects.

### Intervention adherence and fidelity

At the start of the programme, rules on attendance are communicated to the patient, and a written contract signed by the patient. Based on the mid-trajectory evaluation, the treatment may be ceased in the case of insufficient attendance or participation. In case the treatment is stopped prematurely, the reason for this stop is recorded in the electronic patient file. This allows for discrimination between drop-outs due to early successful treatment outcome, external factors, insufficient attendance or participation or adverse events.

### Modifications

Due to the onset of the COVID-19 pandemic, between March 30th and May 11th 2020, the treatment was provided entirely remotely through video calls and webcasts. Subsequently, it was decided to keep the webcasts as a regular treatment component (*see above*).

## Limitations

The *CIR Excellent* IMPT involves many different therapy elements and care providers of different disciplines and is individualised to address patient-specific needs and goals. The complex nature of this treatment means that an attempt at a systematic description comes with limitations. Although the framework of the treatment and the therapy elements of which it consists can be described, it is not possible to provide an exact, identically replicable description. In order to cover all important aspects of the treatment, it was necessary to deviate from the standard format of the TIDieR-checklist and adapt it to include the eligibility criteria and evaluation procedures.

## Data Availability

Data sharing is not applicable to this article as no datasets were generated or analysed during the current study.

## Abbreviations

ACT: Acceptance and commitment therapy
CIR: Centre for integral rehabilitation, or dutch: centrum voor integrale revalidatie
CMP: Chronic musculoskeletal pain
EAET: Emotional awareness and expression therapy
EMDR: Eye movement desensitization and reprocessing
GPE: Global perceived effect
IMPT: Interdisciplinary multimodal pain treatment
PHODA: Photograph series of daily activities
PNE: Pain neuroscience education
TIDieR: Template for intervention description and replication
VAS: Visual analogue scale
